# Cardiac Arrhythmias in Oncological Patients—Epidemiology, Risk Factors, and Management within the Context of the New ESC 2022 Guidelines

**DOI:** 10.1007/s11912-023-01445-x

**Published:** 2023-08-17

**Authors:** Michał Gawlik, Jakub Michal Zimodro, Aleksandra Gąsecka, Krzysztof J. Filipiak, Sebastian Szmit

**Affiliations:** 1 1st Chair and Department of Cardiology, Medical University of Warsaw, Banacha 1a, 02-097 Warsaw, Poland; 2 Institute of Clinical Sciences, Maria Skłodowska-Curie Medical Academy in Warsaw, Warsaw, Poland; 3 Department of Cardio-Oncology, Centre of Postgraduate Medical Education, Institute of Hematology and Transfusion Medicine, Warsaw, Poland; 4 Clinic of Oncological Diagnostics and Cardio-Oncology, Maria Skłodowska-Curie National Research Institute of Oncology, Warsaw, Poland

**Keywords:** Arrhythmia, Cardio-oncology, Cardiotoxicity, Cardiovascular disease

## Abstract

**Purpose of Review:**

To provide an update on epidemiology, risk factors, and management of cardiac arrhythmias in oncological patients within the context of the new European Society of Cardiology 2022 guidelines on cardio-oncology.

**Recent Findings:**

One of the side effects of different chemotherapeutics is their pro-arrhythmic activity. Both atrial and ventricular arrhythmias may be induced by cancer itself or by anticancer treatment. Recent studies report on the cardiotoxic activity of such promising therapies as BRAF and MEK inhibitors, or CAR-T therapy.

**Summary:**

Risk factors of arrhythmias in oncological patients overlap with cardiovascular diseases risk factors, but there are some groups of anticancer drugs that increase the risk of cardiotoxicity. It is crucial to be aware of the risks associated with the oncological treatment and know how to act in case of cardiotoxicity.

## Introduction

Cardiovascular diseases (CVD) and cancer are the two most common causes of death worldwide, and their prevalence constantly increases. In 2014, cancer accounted for 1,345,680 deaths in Europe, whereas in 2019 it was responsible for over 1,409,700 deaths [1]. On the other hand, CVD causes over 4,000,000 deaths in Europe per year, accounting for 45% of overall deaths [2]. In August 2022, the first-ever official guidelines of the European Society of Cardiology (ESC) on cardio-oncology were presented. The new guidelines aim to standardize the management and facilitate the care of oncological patients who are exposed to the cardiotoxic effects of anti-cancer treatment. Currently, there is a trend of increasing life expectancy and thus an increased number of elderly people in the population with a higher chance of developing cancer and CVD, such as arrhythmias. The most common arrhythmia observed in oncological patients, as it is in patients without diagnosed cancer, is atrial fibrillation (AF). Other types, such as QT-prolongation and ventricular arrhythmias (VA) or bradyarrhythmias can also appear, but less frequently. In this manuscript, we discuss (i) the epidemiology of the most common arrhythmias resulting from the presence of cancer or anti-cancer treatment, (ii) risk factors for arrhythmia in oncological patients, (iii) the management of various types of arrhythmias in the light of the latest ESC guidelines.

## Epidemiology

The prevalence of arrhythmias in oncological patients differs, depending on the cancer type, oncological treatment, patient’s characteristics, and risk factors. The epidemiology of different arrhythmias in cancer patients is presented below.

### Atrial Fibrillation

The overall incidence of AF in general population varies from 1 to 2%, whereas among oncological patients, it reaches between 5% and 16%, depending on the risk factors and cancer type [3, 4]. Korean Nationwide Population-Based Study showed that AF is strongly connected with the presence of hematological and intrathoracic cancers with HR = 2.69 for esophageal cancer and HR = 2.39 for lung cancer [5•, 6]. In a group of 4,324,545 patients, 316,040 (7.3%) of whom had a cancer diagnosis, the incidence of AF was increased in all cancer types, in comparison to patients without cancer and reached 17.4 per 1000 person-years (PY) and 3.7 per 1000 PY, respectively, during 12 years of follow-up [7]. The highest incidence was in male patients with lung cancer (58.7 per 1000 PY in men and 35.3 per 1000 PY in women). Several clinical trials showed that in patients treated with ibrutinib (Bruton’s kinase inhibitor), the incidence of AF reaches up to 16% [8•, 9, 10]. The analysis of WHO pharmacovigilance database showed 19 cancer drugs significantly associated with the occurrence of AF. They accounted for a total of 6147 out of 11,757 all reported AF cases. Lenalidomide caused 1733 (14.7%), ibrutinib 1431 (12.17%), and docetaxel 395 (3.36%) of AF cases [11].

### QT Prolongation and Ventricular Arrhythmias

The incidence of VAs is smaller than in the case of atrial arrhythmias. However, the severity of the complications after the possible cardiac arrest is more serious. Several groups of medicines are known for prolonging QT, including antiarrhythmics, antidepressants, antifungals, or antiemetics. Other risk factors increasing the risk of VAs in cancer patients are electrolyte abnormalities and inadequately adjusted doses of renal or hepatic-cleared QT-prolonging drugs. All these risk factors will be discussed later in the article.

A retrospective study was conducted to assess the risk of developing VA in patients with implantable cardioverter-defibrillator (ICD) in primary or secondary prevention and their risk of developing VA. A total of 1598 patients were included in the study of whom 209 (13.1%) had malignancy and 102 patients (6.4%) were diagnosed with cancer after ICD was implanted. The most common malignancies in the group of patients enrolled in the study were skin cancers (25%, [*n* = 53]), prostate cancer (12%, [*n* = 26]), breast cancer (12%, [*n* = 25]), lung cancer (9%, [*n* = 18]). Twenty-eight (13%) patients were diagnosed with more than one malignancy. Patients’ history data were followed from January 2007 up to June 2015. The incidence of ventricular tachycardia (VT) or ventricular fibrillation (VF) was at the level of 39.6% in cancer patients with ICDs in the primary prevention and 56% in the secondary prevention. The incidence was nearly 10 times higher in patients with cancer, reaching 1.19±0.32 episodes per month, compared to the patients without oncological disease—0.12±0.21 [12, 13••]. The ischemic cause of VA was confirmed in 43.9% of the incidents.

BRAF and MEK inhibitors (BRAFi/MEKi) are used in the pharmacological treatment of BRAF600 mutant melanoma. About 50% of metastatic cutaneous melanomas have this mutation, which causes dysregulation in the RAF-MEK-ERK pathway. Inhibiting this pathway results in cell proliferation control and thus melanoma control. BRAFi/MEKi cause cardiotoxicity probably due to overlapping between pathogenic cancer pathways and pathways needed for normal cardiac physiology [13••]. A multicenter study was conducted with 371 patients, who received vemurafenib (BRAFi) treatment [14]. It showed QT interval prolongation in 9.5% (*n* = 35). Twenty-four patients had QTc interval > 480ms, while 11 patients had QTc prolongation to more than 500ms. QTc interval prolongation of at least 60ms was registered in 19 patients (11%). QT prolongation is mainly the problem in the treatment with BRAFi, but it is not observed in the case of dabrafenib and encorafenib [15, 16].

### Bradyarrhythmias

Bradycardia is defined as a heart rate below 60 beats per minute. It is rather rare in oncological patients. The majority of bradyarrhythmia episodes are asymptomatic. Fortunately, there is rarely a need for adjusting the drug dose or other interventions.

The best-known group of chemotherapeutics that is likely to cause bradycardia are anaplastic lymphoma kinase (ALK) inhibitors, for instance, crizotinib or ceritinib. They are used mainly in non-small cell lung cancer (NSCLC) treatment [3]. A retrospective analysis of 1053 patients was conducted, including patients who had at least one pretreatment heart rate (HR) measurement and afterwards were given crizotinib in advanced NSCLC therapy. A total of 41.9% (441) patients experienced at least one recorded episode of sinus bradycardia (SB). The mean maximum decrease of HR was 30.0 bpm. It was higher than in patients who did not experience sinus bradycardia, where the mean maximum decrease of HR was 21.4 bpm; 5.9% of 441 patients experienced the lowest HR below 45 bpm. The majority of patients (75.3%) had the lowest recorded HR of 50 to 59 bpm [17].

Altogether arrhythmias appear more frequently in the population of oncological patients. All types of cancers increase the risk of developing arrhythmia, but that risk can be lowered by changing the drug dose, type of therapy, or modifiable risk factors.

## Risk Factors of Arrhythmias in Oncological Patients

Many risk factors are increasing the chance of arrhythmia in oncological patients. Some of them may overlap with risk factors for CVD, for instance, advanced age, race, or endocrine and metabolic disorders. There are also numerous groups of medicines used in cancer treatment, which are known for their pro-arrhythmogenic effect. The risk factors can be divided into four categories:

- non-chemotherapeutic factors (prior arrhythmogenic substrate, post-surgery arrhythmia, arrhythmogenic medications i.a. antiemetics);
- cardiotoxicity of chemotherapeutic treatment;
- direct cardiac involvement (primary cancer of heart, metastasis to heart);
- electrolyte abnormalities (as a result of vomiting or drug-induced).

### Risk Factors of Atrial Fibrillation

As in the non-cancer population, age is the main risk factor for AF with a steep increase after the age of 65. In the USA, 40.3% of population is predicted to have some kind of CVD [18]. In addition, there are also ethnic differences in the incidence of AF. It is lower in the population of Asians and Afroamericans than in the European population [19]. There are also modifiable risk factors, which include general cardiovascular risk factors like hypertension, obesity, or diabetes mellitus. With proper management, patients can lower the risk of developing AF. In patients with cancer that risk is higher, regardless of the type of cancer, but the highest incidence is observed in the group of patients with lung cancer. Predominantly AF occurs within 0–90 days from the cancer diagnosis [7]. Autonomic nervous system imbalance can be observed in oncological patients. It is mainly because of the chronic inflammation, pain, and other types of stress associated with cancer treatment. Chemotherapeutics associated with the highest incidence rate of occurring AF are for instance anthracyclines, tyrosine kinase inhibitors (TKIs), fluoropyrimidines, or melphalan.

#### Anthracyclines

Anthracyclines are used in the treatment of many different types of cancers, for instance, pediatric solid malignancies, breast cancer, or gynecologic malignancies. Unfortunately, they are known for their irreversible cardiotoxic acting causing cardiomyopathy and arrhythmias. It is known that acute cardiotoxicity causes possibly reversible arrhythmias after discontinuation, whereas late cardiotoxicity can lead to cardiomyopathy, valvular damage, and more serious arrhythmias [20, 21]. Doxorubicin may suppress the expression of Ca2+ ATPase in sarcoplasmatic reticulum (SR), which can impair calcium regulation and in consequence heart function [22]. Doxorubicin activates calmodulin kinase II (CAMKII), which results in Ca2+ leakage from the SR mediated by CAMKII [23]. In this mechanism, it may increase the incidence rate of AF.

#### Tyrosine Kinase Inhibitors

TKIs are used in the treatment of chronic lymphocytic and myeloid leukemia (CLL, CML), lung cancer, and colorectal cancer. This group of medicines consists of several subgroups: anti-VEGF TKIs (anti-vascular endothelial growth factor), Bruton’s TKIs, and BCR-ABL TKIs.

Anti-VEGF TKIs (sorafenib, axitinib, or pazopanib) are used in the treatment of kidney cancer advanced renal cell carcinoma (RCC), or hepatocellular carcinoma (HCC). These molecules act at intracellular parts of VEGF receptors. Although VEGF-TKIs improved progression-free and overall survival in patients with RCC they can cause cardiotoxic effects. One of the possible mechanisms is that they act on various signaling pathways, and the less specific they are, the more possible effects they may have. They also inhibit tyrosine kinases, which are normally expressed in tissues like myocardium or blood vessels. Inhibition of VEGF receptors causes vasoconstriction, an increase in oxidative stress, and the repair capacity of cardiomyocytes is decreased. Nevertheless, the exact mechanism of cardiotoxicity of TKIs is not fully understood. A study on major adverse cardiovascular effects (MACE) showed that 2.66% (*n* = 22) patients enrolled in the study, treated with sorafenib, pazopanib, or sunitinib, had rhythm disorders—AF and atrioventricular block [24]. The authors of the study suggest that the occurrence of AF at the early stage of the TKI treatment was caused by increased blood pressure and diastolic dysfunction of the left ventricle.

One of the representatives of Bruton’s TK (BTK) inhibitors is ibrutinib. It is used in the treatment of CLL, marginal cell lymphoma, or mantle cell lymphoma. Ibrutinib acts on cancer cells by inhibiting Bruton’s kinase, but it also shows an off-target effect on TEC protein kinase. A mice model showed that ibrutinib dysregulates atrial calcium channels and can lead to AF by increasing spontaneous Ca2+ release [25]. Ibrutinib shows even a 10-fold increased risk of developing AF after long-term treatment [10, 26, 27].

#### Fluoropyrimidines

Fluoropyrimidines, namely 5-fluorouracil and capecitabine, are widely used as a treatment of solid tumors, such as gastrointestinal and colorectal cancer, breast cancer, or head and neck tumors. A prospective observational trial (CHECKPOINT) included 129 patients eligible for analysis with a histologically confirmed diagnosis of localized or metastatic colorectal cancer [28•]. The patients had no prior treatment with 5-fluorouracil or capecitabine, but they had a clinical indication for such treatment, according to national guidelines [29]. The results showed 20 out of 129 patients (15.5%) experienced fluoropyrimidines induced cardiotoxicity. The most common symptoms were dyspnea (*n* = 12, 60%), chest pain (*n* = 8, 40%), and palpitations (*n* = 8, 40%). Three patients had relevant ECG changes, of whom one had left bundle branch block (LBBB), and one had supra-ventricular paroxysmal tachycardia. The last patient had ST deviation with elevated troponin I levels and was diagnosed with acute myocardial infarction.

#### Melphalan

Melphalan belongs to a group of alkylating agents and is a chemotherapy treatment for different cancer types, like non-resectable epithelial ovarian cancer, and neuroblastoma. It is also used before hematopoietic stem cell transplant (HSCT) as a high-dose conditioning treatment. A retrospective analysis of 1221 patients who underwent HSCT in years from 1998 to 2005 evaluated the incidence and risk factors of supraventricular tachycardia in patients treated with melphalan [30]. Multiple myeloma was the indication in 34.23%, non-Hodgkin lymphoma in 17.5%, breast cancer in 14.82%, and Hodgkin lymphoma in 6.06%. SVT occurred in 62 cases (5.1%) of all patients who were given chemotherapy with the HSCT. Melphalan was most commonly used (*n* = 438) among other chemotherapy regimens; 35 (8%) out of 438 patients who were given melphalan developed AF/AFl, and 13 patients (3%) experienced other types of SVT. The analysis showed the rate of SVT after melphalan treatment was higher than in other chemotherapeutic regimens.

### Risk Factors of Ventricular Arrhythmia

The QT interval shows the duration of ventricular depolarization and repolarization. The upper limit of the normal QTc value is 450 ms for men and 460 ms for women [31]. Prolongation of QTc interval over 500ms is associated with a 3-fold higher risk of developing TdP [32]. The new ESC guidelines on cardio-oncology specify two categories either correctable or non-correctable risk factors of occurring LQTc and VA. The first group includes factors such as QT-prolonging drugs (i.a. antiarrhythmics, antibiotics, antihistamines, antiemetics), bradycardia, and electrolyte imbalance caused by anticancer treatment. The non-correctable group consists of such risk factors as the age above 65 years, female sex, family history of sudden cardiac deaths, congenital long QT syndrome (LQTS), pre-existing abnormal renal function, or liver disease [4]. VAs induced by cancer therapy are mostly associated with prolonging QTc and thus causing TdP. New ESC guidelines provide short formula “AAGNO P-R-S-T-V” to remember 10 most important drugs with high risk of QT interval prolongation (aclarubicin, arsenic trioxide, glasdegib, nilotinib, oxaliplatin, pazopanib, ribociclib, sunitinib, toremifen, vandetanib).

#### Ribociclib

Ribociclib is a cyclin-dependent kinase (CDK) 4/6 inhibitor, used to treat hormone-positive (HR+), locally advanced, or metastatic breast cancer. The safety, pharmacokinetics, and pharmacodynamics of escalating doses of ribociclib were investigated in a phase I study, which included 132 patients with solid tumors or lymphomas, with intact retinoblastoma protein (Rb+) [33]. The median exposure to the study treatment was 8 weeks. The purpose of the study was to determine the maximum tolerated dose (MTD), dose ready for expansion (RDE), dose-limiting toxicities (DLTs), pharmacokinetics, pharmacodynamics, and safety of the treatment. MTD was estimated at the level of 900mg/d. The RDE was determined as 600mg/d, because of the lower rate of QTc prolongation, compared to the higher doses. QT interval prolongation occurred in 9% of cases. It was always asymptomatic and reversible.

#### Arsenic Trioxide (ATO)

ATO is a chemotherapeutic used in the treatment of relapsed, as well as in newly diagnosed acute promyelocytic leukemia (APL). Many clinical trials showed a prolongation of QT interval. A multicenter study conducted in the USA showed that 25 (63%) out of 40 patients enrolled in the study had significant QT prolongation, as ECG was performed. One of the participants had a QT interval >500ms and had a short, asymptomatic run of torsade de pointe [34].

#### Thyrosine Kinase Inhibitors—Sunitinib

Sunitib is a chemotherapeutic belonging to TKIs. It is used in the treatment of solid tumors like renal cell carcinoma (RCC), gastrointestinal stromal tumor, or pancreatic neuroendocrine tumors. The most common adverse effects (AEs) of sunitinib are kidney failure, heart failure, and hematological complications. Sunitinib can also cause QT interval prolongation and lead to VA, which was shown in the following study [35]. Forty-eight patients were enrolled in the study, and 44 patients (92%) received all planned doses of sunitinib. The follow-up duration was 10 days from the initiation of the treatment. Twenty-four patients completed all blood tests assessing pharmacokinetics and ECG tests, and their results could be analyzed. Two patients had grade 1 QTc prolongation (>450–470ms), and the other two patients had grade 2 QTc prolongation (>470–500ms). The study showed sunitinib’s dose-dependent effect on QTc prolongation. There was an increase in QTc along with increasing drug concentration. The benefits of sunitinib treatment should be weighed against the risk of potential VA in oncological patients.

### Risk Factors of Bradyarrhythmia

**Fig. 1:**
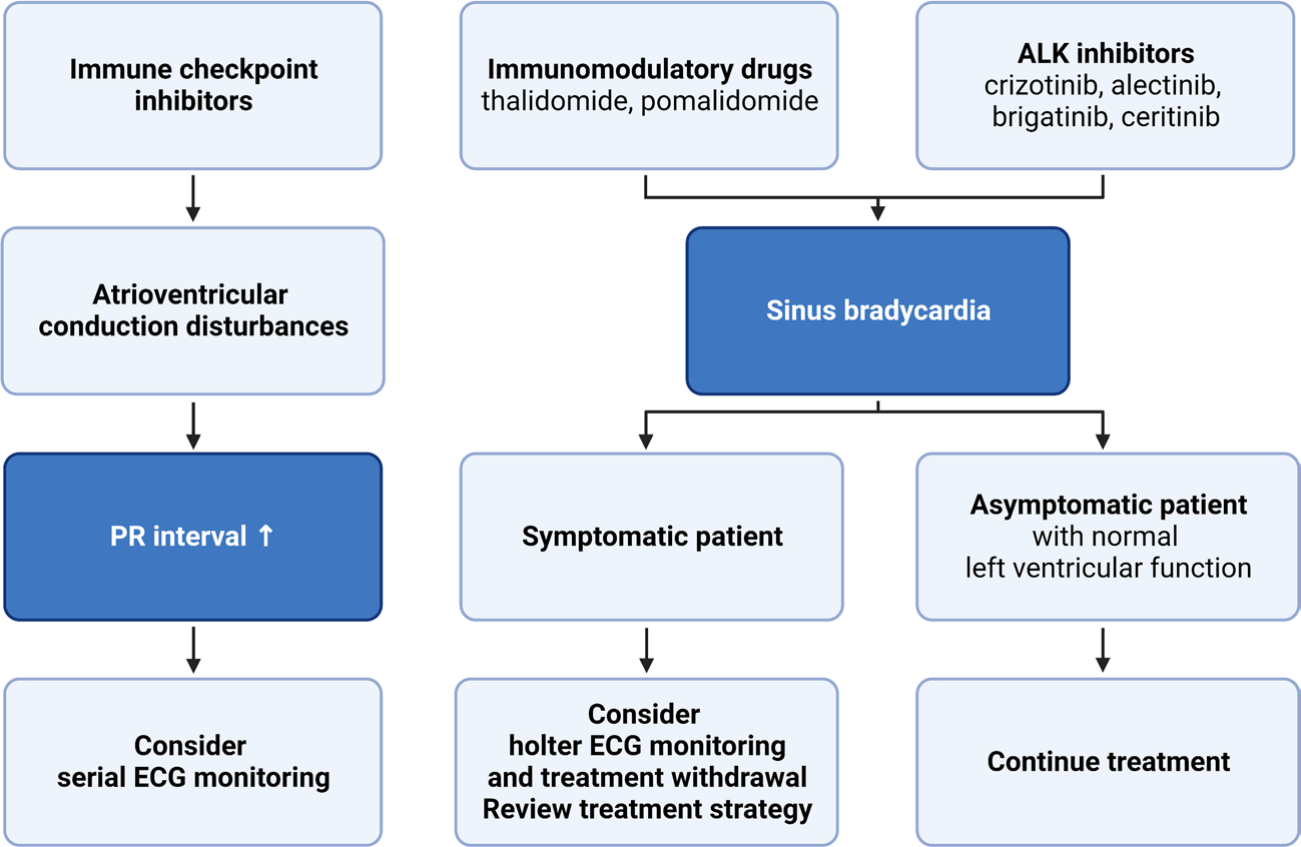
Mechanisms of drug-induced bradyarrhythmias in cancer patients [38–41]

Bradyarrhythmic events can be easily underdiagnosed because they are often asymptomatic. There are several risk factors for bradycardia. It can occur secondary to the neck tumor with the involvement of the vagus nerve, secondary to the infiltration of the atrioventricular node by cancer, or as a side effect of oncological treatment. Among the drugs listed as possible risk factors for bradycardia are antimetabolites (5-fluorouracil, cytarabine), ALK inhibitors (crizotinib—the highest risk). The mechanisms of drug-induced bradyarrhythmias in cancer patients are shown in Fig. 1.

#### Antimetabolites

Antimetabolites, including 5-fluorouracil and citarabine, are thought to be the third most commonly used anticancer treatment in solid malignancies. Yet, they are not free from drug-related toxicities. After anthracyclines antimetabolites are the group most frequently causing cardiotoxicity. A case of a 28-year-old patient showed the relation between cytarabine and bradycardia. He was admitted to the emergency department, where he was diagnosed with acute myeloid leukemia. He was qualified and received treatment with the 3 + 7 protocol with idarubicin and cytarabine. As he experienced dizziness with no other symptoms, he was found to have bradycardia with an HR of 30 to 40 beats per minute [36].

#### Anaplastic Lymphoma Kinase Inhibitors (ALK Inhibitors)

Another group of medicines associated with an increased risk of developing bradycardia is ALK inhibitors, such as crizotinib, ceritinib, or alecitinib widely used in the treatment of NSCLC. A retrospective analysis was made to assess the risk of sinus bradycardia in patients treated with alecitinib [37]. Between the years 2020 and 2022, 93 patients with ALK-positive NSCLC diagnosis and who were treated with alecitinib were analyzed. The patients had HR recorded before and after drug administration. It turned out that 50.54% of patients had at least one episode of sinus bradycardia. In 61.7% of cases, it was developed within 3 months after alecitinib was first administrated.

Apart from the treatment with chemotherapeutics bradycardia can also be associated with high-dose thoracic radiation therapy [38].

### Immunotherapies as a Risk Factor for Arrhythmias in Cancer Population

Recent studies on the cardiotoxicity of chemotherapeutic treatment suggest that immunotherapies, like immune checkpoint inhibitors or chimeric antigen receptor T-cell therapy (CAR-T), may cause arrhythmias.

Immune checkpoint inhibitors (ICIs) are used to treat for instance melanoma or lung cancer. They are a type of monoclonal antibody agents that activate patients’ immune system to kill cancer cells. A large analysis included 5518 patients treated with ICIs [39•]. Within 1 year from the beginning of the therapy 12.5% (*n* = 691) of patients developed cardiotoxicity. The most common cardiac AE was newly diagnosed arrhythmia (9.3%), followed by myocarditis (2.1%) and acute myocardial infarction (1.7%). Patients treated with ipilimumab or pembrolizumab had higher risk of developing cardiac AEs (aHR: 2.00; 95% CI: 1.49–2.70; *p* < 0.001; aHR: 1.21; 95% CI: 1.01–1.46; *p* = 0.040 respectively) comparing to those treated with nivolumab. CTLA4 inhibitors had the highest risk of occurring of the cardiotoxicity in comparison with other classes of ICIs (aHR: 1.77; 95% CI: 1.35–2.34; *p* < 0.001). The most common arrhythmia associated with the use of ICIs is AF with the incidence of 4.6% [40].

CAR-T therapy is a revolutionary treatment method. It is approved to treat some kinds of leukemias, lymphomas, and multiple myeloma. One of the AEs is cardiotoxicity. A total of 137 patients, who received CAR-T between January 2016 and November 2018, were included in the retrospective cohort study to evaluate possible cardiac toxicities. The most common indications for CAR-T among enrolled patients were relapsed, diffuse large B-cell lymphoma (61%), and transformed follicular lymphoma (27%). New onset arrhythmia (SVT, AF) was observed in 12% (*n* = 7) [41].

Predominantly, risk factors of arrhythmias in oncological patients overlap with the risk factors of CVD, such as age, and metabolic and endocrine disorders. However, there are some arrhythmogenic groups of chemotherapeutics, like ATO, melphalan, or sunitinib that predispose to a specific type of arrhythmia. Others, like anthracyclines, are known for their high cardio-toxic potential and can cause different conduction disorders.

## Management of Arrhythmias in Oncological Patients

### Atrial Fibrillation and Supra-ventricular Tachycardia

The new guidelines on cardio-oncology suggest that the management of patients with cancer and concomitant AF should follow current ESC Guidelines for diagnosis and management of AF, with the use of the integrated ABC pathway (anticoagulation/avoid stroke, better symptom control, cardiovascular risk factors, and concomitant diseases management). They also propose a new T-B-I-P algorithm (thromboembolic risk, bleeding risk, drug–drug interactions, patient preferences) to assess the risk of anticoagulation in patients with cancer and concomitant AF. The risk of stroke and systemic embolism should be evaluated with the CHA2DS2-VASc score, although it is not fully validated for use in oncological patients. Long-term anticoagulation must be considered in men with ≥1 point and women with≥ 2 points and is recommended in men with ≥ 2 points and women with ≥3 points. In patients with severe mitral stenosis or mechanical prosthetic valve vitamin K antagonists (VKA) are the drugs of choice. Low molecular weight heparin (LMWH) can be considered a short-term option in patients freshly diagnosed with cancer, with advanced cancer, or during anticancer treatment. New oral anticoagulants (NOAC) use in oncological patients is very limited, due to multiple drug-drug interactions, although recently made meta-analysis suggests their similar effectiveness compared to VKA treatment in patients with cancer [42]. The data on the usage of the left atrial appendage occluder devices have very limited data among this group of patients. The use of those devices can be associated with an increased risk of complications, such as device-related thrombosis in oncological patients.

The bleeding risk should be evaluated with a HAS-BLED score. The modifiable bleeding-promoting risk factors (for instance thrombocytopenia, recent major bleeding, gastrointestinal cancer diagnosis) should be identified. The next step is assessing drug interactions taking into account anticancer agents as well as other supportive therapies. Finally, patients’ preferences and drug availability should also be taken into account.

### Ventricular Arrhythmias

Patients to be treated with drugs that have a high risk of QTc prolongation should have baseline 12-lead ECG performed and also modifiable risk factors of VA should be corrected. If the baseline QTc interval is ≤ 480ms, the therapy can be started under ECG monitoring. The ECG should be performed once the blood level of the anticancer drug is achieved, after every modification in the treatment, and once a month for the first 3 months. According to current ESC guidelines on cardio-oncology, QTc interval changes above 60ms, with QTc still < 500ms, should not affect oncological treatment.

Patients with QTc > 480ms require closer monitoring. Reversible causes of long QT interval should be corrected, ECGs performed weekly, the risk of VA evaluated during the treatment, and an alternative treatment considered. In patients with abnormalities in baseline QTc interval, patients with symptoms of arrhythmia or treated with QTc prolonging drugs cardiology consultation are advised.

The treatment of anticancer therapy-induced VA should follow general guidelines for the management of VA [32]. Asymptomatic episodes of VA that are self-terminating should not be a reason to terminate the oncological treatment, unless patients have persisting ECG abnormalities. The intervention is required in case of recurrent VA. However, the usage of anti-arrhythmic drugs is limited, due to drug-drug interactions and possible further QTc prolongation. Current ESC guidelines recommend beta-blockers and class IB antiarrhythmics as the safest option in the VA treatment, because they are less likely to interact with other drugs. Beta-blockers are the drugs of choice when cancer drug is known to cause cancer therapy-related cardiac dysfunction. Amiodarone is preferred in patients, who have a structural cardiac disease or that are hemodynamically unstable. Decisions on the type of therapy should be made on an individual basis and should take into account factors, like complication risk or predicted life expectancy.

### Bradycardia

Bradycardia episodes secondary to cancer treatment are usually asymptomatic depending on the heart rate at the time of the episode [43]. Possible symptoms include dizziness, fatigue, pre-syncope, or syncope. If the patient is symptomatic, Holter-ECG is recommended to assess the severity of the bradycardia and exclude long sinus pauses. A trial of withdrawing anticancer drugs should be performed to confirm the coincidence with the symptoms. A multidisciplinary team should discuss the risks and benefits of continuing the treatment at a lower dose and eventually consider alternative therapy. The pacemaker implantation is indicated, when there is no substitution for the current treatment. The final decision should take into account patients’ preferences and should be made after cardiologist and oncologist consultation.

To summarize, the management of arrhythmias in oncological patients should be based on current clinical practice guidelines, especially the newest ESC Guidelines on Cardio-oncology. Careful patient observation for possible symptoms of the arrhythmia and ECG monitoring are the basis of the management during oncological treatment with high arrhythmogenic potential.

## Conclusions

Arrhythmias in oncological patients occur more frequently than in the general population. This trend is observed in all cancer types. The incidence depends on the patient’s characteristics, risk factors, as well as cancer’s grade and stage. Many anticancer treatments are major risk factors for arrhythmias, for instance, anthracyclines, antimetabolites, or TKIs. Also, the newest studies show that immunotherapy is associated with cardiotoxicity. In the management of oncological patients, it is crucial to follow the guidelines for diagnostics and management of specific arrhythmias, taking into account both the classical factors, such as prior arrhythmogenic substrates, and the specific oncological problems, including cardiotoxicity of chemotherapeutic treatment, direct cardiac involvement, and electrolyte abnormalities.
